# Structural Insights into Substrate Recognition by *Clostridium difficile* Sortase

**DOI:** 10.3389/fcimb.2016.00160

**Published:** 2016-11-22

**Authors:** Jui-Chieh Yin, Chun-Hsien Fei, Yen-Chen Lo, Yu-Yuan Hsiao, Jyun-Cyuan Chang, Jay C. Nix, Yuan-Yu Chang, Lee-Wei Yang, I-Hsiu Huang, Shuying Wang

**Affiliations:** ^1^Department of Microbiology and Immunology, College of Medicine, National Cheng Kung UniversityTainan, Taiwan; ^2^Institute of Bioinformatics and Structural Biology, National Tsing Hua UniversityHsinchu, Taiwan; ^3^Bioinformatics Program, Taiwan International Graduate Program, Academia SinicaTaipei, Taiwan; ^4^Department of Biological Science and Technology, National Chiao Tung UniversityHsinchu, Taiwan; ^5^Molecular Biology Consortium, Advanced Light Source, Lawrence Berkeley National LaboratoryBerkeley, CA, USA; ^6^Physics Division, National Center for Theoretical SciencesHsinchu, Taiwan; ^7^Center of Infectious Disease and Signaling Research, National Cheng Kung UniversityTainan, Taiwan

**Keywords:** *Clostridium difficile*, sortase, substrate specificity, crystal structure, fluorescence resonance energy transfer

## Abstract

Sortases function as cysteine transpeptidases that catalyze the covalent attachment of virulence-associated surface proteins into the cell wall peptidoglycan in Gram-positive bacteria. The substrate proteins targeted by sortase enzymes have a cell wall sorting signal (CWSS) located at the C-terminus. Up to date, it is still not well understood how sortases with structural resemblance among different classes and diverse species of bacteria achieve substrate specificity. In this study, we focus on elucidating the molecular basis for specific recognition of peptide substrate PPKTG by *Clostridium difficile* sortase B (Cd-SrtB). Combining structural studies, biochemical assays and molecular dynamics simulations, we have constructed a computational model of Cd-SrtB_ΔN26_–PPKTG complex and have validated the model by site-directed mutagensis studies and fluorescence resonance energy transfer (FRET)-based assay. Furthermore, we have revealed that the fourth amino acid in the N-terminal direction from cleavage site of PPKTG forms specific interaction with Cd-SrtB and plays an essential role in configuring the peptide to allow more efficient substrate-specific cleavage by Cd-SrtB.

## Introduction

Bacterial surface proteins are crucial virulence factors that mediate adhesion to the host as the first step establishing an infection. The sortase family of cysteine transpeptidases catalyzes the anchoring of a wide variety of virulence-associated surface proteins to the cell wall peptidoglycan (Spirig et al., 2011; Cascioferro et al., 2014; Bradshaw et al., 2015). Sortases, unique to Gram-positive bacteria, recognize and cleave the C-terminal cell wall sorting signal motif (CWSS) of substrate proteins (Schneewind et al., 1992, 1993; Paterson and Mitchell, 2004). Based on the primary sequences and their roles in biological functions, sortases are classified into six classes: A, B, C, D, E, and F. Class A sortases (SrtAs) are present in almost all Gram-positive bacteria. The first identified and best-known class A enzyme is the *Staphylococcus aureus* SrtA (Sa-SrtA), which anchors at least 19 LPXTG-containing surface proteins (Mazmanian et al., 1999; Perry et al., 2002; Spirig et al., 2011; Bradshaw et al., 2015). Sa-SrtA mutants exhibited a severe reduced adherence to epithelial cells and virulence in animal models (Flock et al., 1987; Mazmanian et al., 2000; Clancy et al., 2010). SrtAs are commonly called housekeeping sortases, whereas the remaining five classes are the accessory sortases. Class B sortases (SrtBs) recognize the NXXTN motif rather than the classical LPXTG motif and have distinct functions (Comfort and Clubb, 2004; Dramsi et al., 2005); some members of this group are involved in iron acquisition, whereas sortase B of *Streptococcus pyogenes* is involved in pili assembly (Kang et al., 2011). Class C sortases (SrtCs) are essential for pili polymerization in many species (Huang et al., 2010), such as *Enterococcus faecalis* (Kline et al., 2009), *Corynebacterium diphtheria* (Ton-That and Schneewind, 2003; Gaspar and Ton-That, 2006), *Streptococcus agalactiae* (Dramsi et al., 2006; Cozzi et al., 2012), and *Streptococcus pneumonia* (Fälker et al., 2008; LeMieux et al., 2008; Manzano et al., 2008). In addition, SrtC is required for aerial hyphae formation in *Streptomyces coelicolor* (Di Berardo et al., 2008). Class D sortases (SrtDs) are similar to SrtAs and perform a housekeeping role; they most frequently present in *Bacillus* species and are involved in spore formation (Marraffini and Schneewind, 2006). Recent studies have reported that *Clostridium perfringens* SrtD is structurally and catalytically distinct from *Bacillus anthracis* SrtD, suggesting that *C. perfringens* SrtD may display a different aspect of the SrtD family (Marraffini and Schneewind, 2006; Suryadinata et al., 2015). Class E and F sortases are mainly identified in *Actinobacteria*; they share a limited primary sequence homology with other sortases and their functions remain undetermined (Comfort and Clubb, 2004; Dramsi et al., 2005; Spirig et al., 2011).

In the genome of toxigenic *C. difficile* strain 630, only one functional sortase, the SrtB gene, is present (Donahue et al., 2014). *C. difficile* is a Gram-positive, anaerobic, and spore-forming bacterium that can colonize the gut if the normal intestinal microbiota is disrupted (Kelly and LaMont, 1998). *C. difficile* infection (CDI) is highly associated with antibiotic therapy and has been recognized as the leading cause of antibiotic-associated diarrhea, making it a major public health threat worldwide (Henrich et al., 2009; Bagdasarian et al., 2015). In the United States alone, CDI causes approximately 15,000–20,000 deaths annually, and CDI-associated hospitalizations among the general population doubled from 31 to 61 per 100,000 from 2008 to 2010 (Viseur et al., 2011). Furthermore, the CDI risk is high in patients receiving antibiotic treatments because their gastrointestinal flora is unfavorably altered. CDI manifestations can include asymptomatic colonization, mild to severe chronic diarrhea, pseudomembranous colitis, and death because of multiple organ failure (Kelly and LaMont, 2008). At present, metronidazole and vancomycin are mainly administered for treating CDI. However, up to 25% of patients treated for CDI experience recurrences after discontinuing antibiotic therapy (Bartlett et al., 1980; Tedesco et al., 1985; Leffler and Lamont, 2009; Surawicz et al., 2013). The increase in treatment failure or multiple relapses have raised a concern. An alternative therapy, fecal microbiota transplantation, has been used to restore healthy gut flora in patients with recurrent CDI (Rohlke and Stollman, 2012; Dodin and Katz, 2014; Duke and Fardy, 2014). Fecal transplantation is highly effective; however, it is still not widely accepted. In the last decade, sortase has been identified as a promising anti-infective therapeutic target (Zong et al., 2004b; Maresso et al., 2007; Suree et al., 2009; Oh et al., 2010; Jacobitz et al., 2014; Zhang et al., 2014), thus offering an encouraging avenue toward the development of drugs against CDI.

There have been many structural and functional studies on sortases from various Gram-positive pathogens (Spirig et al., 2011; Cascioferro et al., 2014; Bradshaw et al., 2015), and studies on *C. difficile* sortases were reported recently (Donahue et al., 2014; van Leeuwen et al., 2014; Chambers et al., 2015). Sa-SrtA has been extensively studied, and the catalytic mechanism underlying how Sa-SrtA anchors the surface protein to cell wall has been reported (Mazmanian et al., 1999; Perry et al., 2002). The membrane-bound Sa-SrtA scans and recognizes the LPXTG sequence of the CWSS, and a nucleophilic attack from the active thiol group of sortase cysteine residue to the peptide bond between the threonine and glycine of the LPXTG motif results in the formation of a thioester intermediate (Mazmanian et al., 1999; Ton-That et al., 1999; Perry et al., 2002). The sortase–acyl intermediate is then resolved by the nucleophilic attack of a free amino group within lipid II, resulting in the release of the surface protein from the sortase onto the cross bridge of the newly formed peptidoglycan (Frankel et al., 2005). This substrate release restores the enzyme active site, allowing the sortase to process more substrates (Frankel et al., 2005).

Recent studies have demonstrated that *C. difficile* SrtB (Cd-SrtB) can recognize and cleave (S/P)PXTG between threonine and glycine; however, Cd-SrtB cannot recognize the sequence LPXTG and NPQTN, corresponding to the recognition motifs for Sa-SrtA and Sa-SrtB, respectively (van Leeuwen et al., 2014; Chambers et al., 2015). It remains unclear how structurally similar sortases achieve substrate specificity. Thus far, the available structures of sortase–substrate complexes are limited to the nuclear magnetic resonance structure of Sa-SrtA bound to an LPATG substrate analog (Suree et al., 2009), a crystal structure of a Sa-SrtA mutant complexed with LPETG (Zong et al., 2004a), and a crystal structure of Sa-SrtB covalently bound to an NPQTN analog (Jacobitz et al., 2014). Therefore, studying and comparing new structures of sortases and sortase–substrate complexes from a wide range of organisms will enhance our understanding of how sortases recognize their respective substrates. In this study, we determined the crystal structure of the catalytically active SrtB from *C. difficile* and constructed a probable model of the Cd-SrtB–PPKTG complex by computer modeling and molecular dynamics simulations to gain structural insights into the substrate specificity for Cd-SrtB.

## Materials and methods

### Protein overexpression and purification

The SrtB_ΔN26_ from *C. difficile* 630 was cloned into a pMCSG7 vector by using a ligation-independent cloning method (Aslanidis and de Jong, 1990) and transformed into *E. coli* BL21 (DE3). A recombinant 6xHis-tagged SrtB_ΔN26_ protein was induced by adding 0.5 mM isopropyl-β-D-thiogalactopyranoside when the cells reached an O.D._600_ of 0.5, and further incubated at 37°C for 4 h. Cells were centrifuged at 8000 rpm for 30 min at 4°C, resuspended in buffer A (20 mM HEPES pH 7.4, 200 mM NaCl and 20 mM imidazole), and disrupted by sonication on ice. Moreover, the supernatant was loaded into an Ni-NTA column (GE Healthcare Life Sciences) and contaminant proteins were eliminated through a washing procedure by using 60 mM imidazole in buffer A. SrtB_ΔN26_ proteins were eluted with 300 mM imidazole in buffer A. Fractions containing SrtB_ΔN26_ proteins were pooled and further purified through HiLoad 26/600 Superdex™ 75 size-exclusion chromatography (GE Healthcare Life Sciences). Subsequently, the proteins were dialyzed in buffer B (10 mM HEPES pH 7.4 and 150 mM NaCl) and stored at 4°C for further use.

### FRET-based assay

The peptide substrate of Cd-SrtB_ΔN26_, PPKTG was conjugated using a fluorophore, 5-[(2-aminoethyl) amino] naphthalene-1-sulfonic acid, and a quencher, 4-([4-(dimethylamino) phenyl] azo) benzoic acid. To determine the suitable concentrations of Cd-SrtB_ΔN26_ and fluorescently labeled PPKTG peptide in the assay, a matrix of various enzyme and substrate concentrations in the total volume of 100 μL in FRET buffer (10 mM HEPES pH 7.4 and 150 mM NaCl) was reacted in a 96-well black polystyrene plate and was incubated at 37°C for 48 h. The fluorescence signal was monitored at an excitation/emission wavelength of 340/490 nm and recorded every hour during the first 8 h and then at 24, 36, and 48 h by using a Spectra-Max M3 plate reader (Molecular Devices). The optimal concentrations of Cd-SrtB_ΔN26_ and fluorogenic peptide used in our reactions are 240 and 20 μM, respectively. Stock solutions of MTSET and AAEK1 were dissolved in the FRET buffer, and curcumin was dissolved in DMSO. Serial dilutions of inhibitors at the millimolar range were added into the FRET buffer. All experiments were conducted in triplicate. The data are presented as means and standard errors. The statistical significance of the inhibitory effect on enzymatic activity was calculated using GraphPad Prism software (GraphPad Software). Two-tailed unpaired Student *t*-tests revealed significant differences between Cd-SrtB_ΔN26_ + PPKTG and Cd-SrtB_ΔN26_ + PPKTG + inhibitors at different concentrations (^*^p ≤ 0.05, ^**^p ≤ 0.01, and ^***^p ≤ 0.001).

### Crystallization

Purified Cd-SrtB_ΔN26_ proteins were concentrated to 8–10 mg/mL for crystallization trials. For sparse matrix screening, numerous commercial kits (Hampton Research and Emeralds BioSystems) were used for performing the crystallization setup of the vapor diffusion method by using a high-throughput platform (Digilab Genomic Solutions). Cd-SrtB_ΔN26_ crystals were observed in sitting drops containing 0.5 μL of protein and 0.5 μL of various crystallization solutions at 25°C within 1 week. Diffraction quality crystals were obtained using the hanging drop method by mixing 1 μL of protein (10 mg/ml in 10 mM HEPES pH 7.4 and 150 mM NaCl) and 1 μL of solution (0.1 M citric acid pH 3.5, 24% PEG 3350 and 0.1 M glycine). Prior to data collection, the crystals were directly mounted on loops from mother liquor and flash-frozen in liquid nitrogen without an additional cryoprotectant treatment.

### X-ray data collection and processing

Diffraction data were collected on beamline BL13B1 of the National Synchrotron Radiation Research Center (NSRRC; Hsinchu, Taiwan) and beamline 4.2.2 of the Advanced Light Source (Berkeley, CA, USA). Most of our sortase crystals did not diffract beyond 3 Å resolution. The best crystal diffracted to 2.67 Å resolution and native data were collected at BL13B1 of the NSRRC. Ninety frames were collected, each with 1° oscillation and were exposed for 30 s at the wavelength of 1.0 Å with the crystal-to-detector distance of 400 mm at a temperature of 100 K. The data were indexed, integrated, and scaled using HKL2000 (Otwinowski and Minor, 1997). The initial data were scaled to 2.67 Å resolution, but the I/σI decreased to 1.67 at the highest resolution shell (2.77−2.67 Å) suggesting that the data were effective at a resolution of approximately 2.8 Å. The crystallographic parameters and data collection statistics were summarized in Table 1.

**Table 1:** **Crystallographic data and refinement statistics**.

**DATA COLLECTION**	
Wavelength (Å)	1.0
Resolution range (Å)	25.9–2.8 (2.9–2.8)^a^
Space group	*I*23
Unit cell dimensions	121.25, 121.25, 121.25, 90, 90, 90
Total reflections	65563
Unique reflections	7031
Redundancy	9.3 (11.1)
I / σI	45.85 (10.4)
Completeness (%)	94.1 (100)
*R*_merge_ (%)	6.5 (25.9)
Wilson B factor (Å^2^)	66.9
**REFINEMENT**	
*R*_work_ (%)	19.71
*R*_free_ (%)	25.23
**Number of atoms**	
Protein	1529
Water	38
***B*-factors (Å^2^)**	
Protein	55.74
Water	50.42
**Rms deviations**	
Bond lengths (Å)	0.0098
Bond angles (°)	1.207
**Ramachandran plot statistics^b^**	
% of residues in favored regions	96.0
% of residues in allowed regions	4.0
% of residues in outlier regions	0.0

a *The values in parenthesis are for the highest resolution bin*.

b *Residues in favored, allowed, and outlier regions of the Ramachandran plot as reported by MolProbity (Chen et al., 2010)*.

### Structure determination and refinement

The crystal structure of Cd-SrtB_ΔN26_ was solved by molecular replacement method by program Phaser-MR (Mccoy et al., 2007) with the structure of Sa-SrtB (PDB 1QWZ) (Zong et al., 2004a) as a search model. Initially, the structure was determined at 3.5 Å resolution, and a polyalanine model was constructed. With the availability of better native datasets at higher resolutions, the model was manually rebuilt using COOT (Emsley et al., 2010) guided by 2*Fo-Fc* and *Fo*-*Fc* density maps. Computational refinement was conducted using REFMAC (Murshudov et al., 2011) and PHENIX (Adams et al., 2010), with 5% of the data flagged for cross-validation. We first carried out the refinement at 2.67 Å resolution, but the statistics were poor. The structural quality was improved when we systematically excluded the weak inflections by truncating data at different resolutions. Iterative model rebuilding and refinement were conducted. The final refinement statistics for the structural model at 2.8 Å resolution were summarized in Table 1. Coordinates and structure factors with the identifier 5GYJ have been deposited in the Protein Data Bank.

### MD simulations

The peptide of sequence NPQC co-crystalized with *S. aureus* SrtB structure (PDB 4LFD) was positioned in the catalytic pocket of *C. difficile* SrtB by superimposing of *S. aureus* SrtB onto *C. difficile* SrtB (Cd-SrtB). It was then mutated into a set of peptides of our interest, including PPKT and NPQT. PPKTG and NPQTN were modeled by adding one more G and N, respectively, in the C-terminus using the package VMD 1.9.2 (Humphrey et al., 1996). We further replaced the P4 residue in PPKTG to give SPKTG and NPKTG. Missing loops of 27–28 (ML), 162–167 (ESDYDY), 210–216 (TYEFDDA), and 225 (I) in the Cd-SrtB were modeled by UCSF CHIMERA (Yang et al., 2012). Cd-SrtB−peptide complexes were solvated in TIP3P water molecules of 8Å thickness in all directions of a rectangular box (Jorgensen et al., 1983). One hundred and fifty millimolar sodium chloride were added as counter-ions to neutralize the system. Energy minimization and explicit-solvent MD simulations were performed by NAMD 2.10 package (Phillips et al., 2005; Huang and MacKerell, 2013) with CHARMM36 force field (Huang and MacKerell, 2013). Non-bonded interactions were carried out using a cut-off distance of 12 Å, with a switching distance of 10 Å. With periodic boundary conditions, the Particle Mesh Ewald method was employed for calculations of electrostatic energy (Darden et al., 1993). The Cd-SrtB, crystal waters and peptides (PPKTG, SPKTG, NPKTG, and NPQTN) were first restrained to the positions reported in the X-ray crystallography and then gradually released, first on the side chains and then the entire peptide and protein. No hydrogen atom is restrained at all time. After a 1.25 ns canonical ensemble (NVT) heating process and a short isothermal–isobaric (NPT) equilibration, the whole system was allowed for a productive run for 9 ns in a NPT ensemble at 310K and 1 atm, respectively controlled by solvent friction and Nosé-Hoover Langevin piston (Feller et al., 1995). MD simulations trajectories are further analyzed by VMD, MDAnalysis toolkit (Michaud-Agrawal et al., 2011) and in-house programs coded in python.

### Contact frequency analysis for the P4 residue of different substrate peptides

To understand the role of the P4 residue in the substrate peptides, we analyzed the contact frequency of the P4 residue of a peptide with Cd-SrtB_ΔN26_. At every frame, a residue in Cd-SrtB_ΔN26_ situating within 4.0 Å from the P4 residue of the substrate peptides is marked as a contact. For each contacting residue, the contacting percentage is defined as the number of frames that the residue is in contact divided by total number of frames in MD simulations.

### Root-mean square fluctuations (RMSF) analysis for peptide residues

To calculate the root-mean square fluctuations (RMSF) for a residue, we first iteratively superimposed the peptides in all MD snapshots to their mean positions by Kabsch's approach (Kabsch, 1976) until the process converges. RMSF of a residue is calculated as $\sqrt{\frac{\sum_{i=1}^{N}\sum_{k=1}^{M}\left(X_{i,k}\;-\;\bar{X_{i}}\right)2}{NM}}$, where *N* is the number of heavy atoms in this residue; *M* is the total number of frames; *X*_*i, k*_ is the *i*-th heavy atom in the *k*-th frame and ${\bar{X}}_{i}\;$ is the mean position for atom *i* over all the frames.

## Results

### Catalytic activity of the recombinant purified Cd-SrtB_ΔN26_

The recombinant 6xHis-tagged *C. difficile* sortase enzyme with a deletion of 26 residues at the N-terminal transmembrane domain, designated as Cd-SrtB_ΔN26_, was overexpressed in *Escherichia coli* BL21 (DE3) and purified using an Ni-NTA affinity column (Supplementary Figure 1A). Size exclusion chromatography revealed that Cd-SrtB_ΔN26_ was eluted at a volume corresponding to an apparent molecular weight of approximately 24 kDa (Supplementary Figure 1B), suggesting that Cd-SrtB_ΔN26_ exists as a monomer in solution.

**Figure 1:**
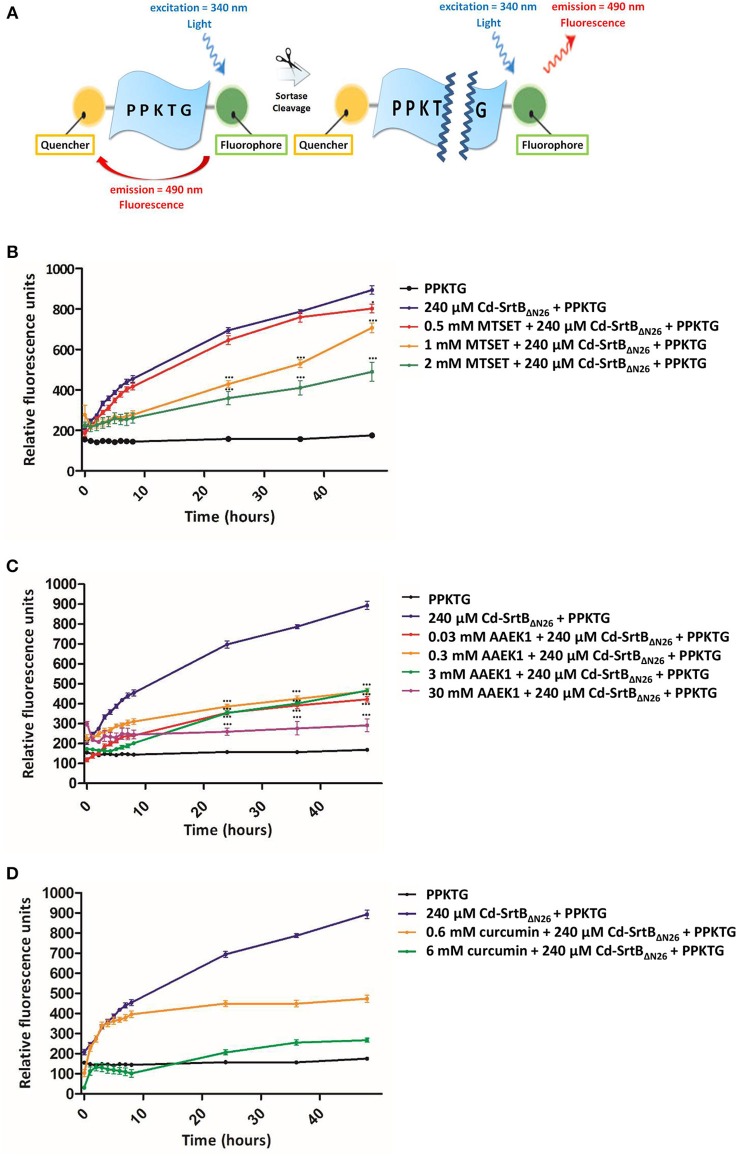
**Enzymatic activity and inhibition of Cd-SrtB_ΔN26_ by using a FRET-based assay. (A)** Schematic representation of the peptide substrate PPKTG sandwiched between Edans and Dabcyl as the fluorophore quencher, respectively. **(B–D)** Catalytic activity of Cd-SrtB_ΔN26_ and the effect of inhibitors monitored using the FRET-based assay. PPKTG was incubated with recombinant purified SrtB_ΔN26_. The increase in the relative fluorescence signal was observed when PPKTG was cleaved by Cd-SrtB_ΔN26_. The enzymatic cleavage of Cd-SrtB_ΔN26_ was inhibited by adding **(B)** 0.5, 1, and 2 mM of MTSET; **(C)** 0.03, 0.3, 3, and 30 mM of AAEK1; and **(D)** 0.6 and 6 mM of curcumin.

To confirm whether the recombinant purified Cd-SrtB_ΔN26_ retains protease activity, we constructed a fluorescently labeled peptide to observe the fluorescence signal after Cd-SrtB_ΔN26_ cleaves the substrate peptide *in vitro* (Figure 1A). In this construct, the known peptide substrate PPKTG (van Leeuwen et al., 2014) is sandwiched between a fluorophore and quencher. When the peptide remains intact, the intrinsic fluorescence is considerably reduced because of the proximity between the fluorescence donor and quenching acceptor. When the peptide is cleaved, the un-quenched fluorophore gives an enhanced fluorescent signal. To assess whether the previously described sortase inhibitors can inhibit the catalytic activity of Cd-SrtB_ΔN26_, MTSET, AAEK1, and curcumin (Maresso et al., 2007; Hu et al., 2013; Donahue et al., 2014) (Supplementary Figure 2) were examined using the fluorescence resonance energy transfer (FRET)-based assay. The concentration-dependent inhibitory effects of MTSET (Figure 1B), AAEK1 (Figure 1C), and curcumin (Figure 1D) on the cleavage activity of the recombinant Cd-SrtB_ΔN26_ were observed as the fluorescence signals were reduced when the inhibitors were added to the reactions comprised of Cd-SrtB_ΔN26_ and fluorogenic peptides. The results further verified the recombinant Cd-SrtB_ΔN26_ is catalytically active.

### Crystal structure of Cd-SrtB_ΔN26_

Cd-SrtB_ΔN26_ comprises 198 residues with a 6xHis tag at the C-terminus. Cd-SrtB_ΔN26_ crystallized in space group *I*23, with the unit cell parameters a = b = c = 121.25 Å and α = β = γ = 90°. The crystal structure of Cd-SrtB_ΔN26_ was solved at 2.8 Å resolution by using the molecular replacement method, revealing one molecule in the crystallographic asymmetric unit. Most of the electron density was visible and interpretable for reliable model building. However, the density map for residues 27 and 28, 162–167, 210–216, and 225 as well as the C-terminal 6xHis tag was disordered. The crystallographic data and refinement statistics are summarized in Table 1. Validation of the Cd-SrtB_ΔN26_ structure by using the MolProbity program (Chen et al., 2010) revealed no phi–psi angles in the disallowed region of the Ramachandran map.

**Figure 2:**
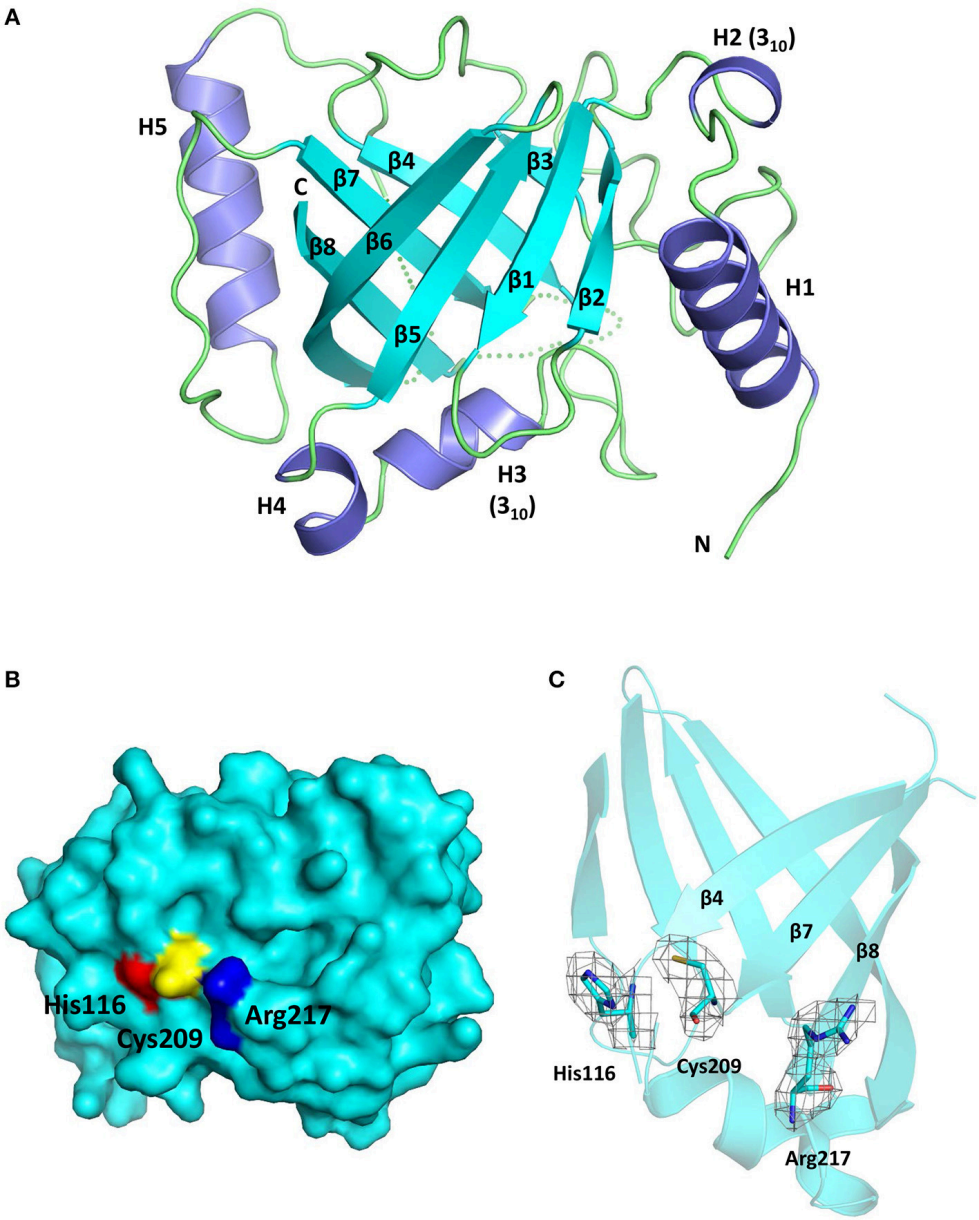
**Crystal structure of Cd-SrtB_ΔN26_. (A)** Ribbon diagram of the SrtB_ΔN26_ structure comprising three α-helices (H1, H4, and H5), two 3_10_-helices (H2 and H3), and eight β-strands (β1–β8). Helices and β-strands are colored in purple and cyan, respectively. The flexible loops are presented in dashes. **(B)** Surface representation of Cd-SrtB_ΔN26_ structure. The catalytic residues Cys209, His116, and Arg217 are colored in yellow, red, and blue, respectively. **(C)** Part of the overall omit map contoured at 1.0 sigma revealing the side-chain density of the catalytic Cys–His–Arg triad.

The Cd-SrtB_ΔN26_ structure possesses the sortase-unique protein fold, comprising eight β-strands (β1–β8), three α-helices (H1, H4, and H5), two 3_10_-helices (H2 and H3), and several loops (Figure 2A). Resembling other sortase structures (Frankel et al., 2007; Kang et al., 2011; Jacobitz et al., 2014), the central β-barrel of Cd-SrtB_ΔN26_ is formed by strands β1, β2, β5, and β6 on one side and by strands β3, β4, β7, and β8 on the other side. The characteristic N-terminal helix bundle, absent in SrtA structures and unique to SrtB, is composed of a 13-residue α-helix (H1), a 3_10_-helix (H2), and a loop. The other 3_10_-helix (H3) and a short α-helix (H4) are positioned between β4 and β5; H5 is inserted between the longest β-strand β6 and β7. The loop connecting β7 and β8 that has been postulated for accommodating peptidoglycan substrate binding was not visible in our structure, implying the flexibility of the large loop. Consistent with previous studies on sortase structures, β4, β7, and β8 of the β-barrel forming the Cys–His–Arg triad of Cd-SrtB_ΔN26_ appears in a concave surface (Figure 2B). The catalytic residues Cys209 and His116 are located slightly beyond the C-terminal ends of β7 and β4, whereas Arg217 is anchored at the beginning of β8 (Figure 2C). A crystal structure of a catalytically inactive *C. difficile* mutant SrtB_ΔN32, C226A_ (PDB 4UX7) was published (Chambers et al., 2015). Superimposition of the catalytic residues of SrtB structures from *C. difficile* (PDB 5GYJ and 4UX7) (Chambers et al., 2015), *S. aureus* (PDB 1NG5) (Zhang et al., 2004), *B. anthracis* (PDB 1RZ2) (Zhang et al., 2004), and *S. pyogenes* (PDB 3PSQ) (Kang et al., 2011) shows the conservation of the active site (Supplementary Figure 3).

Cd-SrtB_ΔN26_ is structurally equivalent to Cd-SrtB_ΔN32, C226A_ (Chambers et al., 2015). However, visualizing the sulfhydryl group of the catalytic cysteine residue in Cd-SrtB_ΔN26_ is essential for facilitating our understanding of the substrate-specific catalysis of Cd-SrtB.

### *In silico* model of the Cd-SrtB_ΔN26_–PPKTG complex

To gain structural insights into how Cd-SrtB_ΔN26_ recognizes PPKTG, we performed computational modeling based on the crystal structure of the Sa-SrtB–NPQT^*^ complex (PDB 4LFD) (Jacobitz et al., 2014) for predicting the Cd-SrtB_ΔN26_–PPKTG structure. In the Sa-SrtB–NPQT^*^ structure, the substrate-binding pocket is delineated by a groove near the active site residues within the strands β4 and β7 and within loops β2/β3, β6/β7, and β7/β8. The NPQT^*^ peptide was bound to Sa-SrtB in an “L-shaped” structure via hydrophobic interactions with Leu96, Tyr128, Tyr181, and Ile182 and via hydrogen bonds with Asn92, Thr177, Glu224, and Arg233. Moreover, the almost superimposable hydrophobic residues from *S. aureus* with the corresponding residues from *C. difficile* underlies the importance of their function (Supplementary Figure 4) and imply that the Cd-SrtB substrate may be positioned in a similar pattern within the hydrophobic groove. Therefore, we superimposed the structure of Sa-SrtB–NPQT^*^ onto Cd-SrtB_ΔN26_, mutated NPQT^*^ to PPKT and added a glycine in the C-terminus *in silico* using the software VMD 1.9.2 (Humphrey et al., 1996) as an initial model of the Cd-SrtB_ΔN26_–PPKTG complex. In addition, the missing residues and loops in the Cd-SrtB_ΔN26_ structure, including the N- and C-terminal residues (27, 28, and 225), and residues located on the β6/β7 (162–167) and β7/β8 (210–216) loops were modeled using UCSF CHIMERA (Yang et al., 2012). To refine the docking pose of the PPKTG in the catalytic pocket of Cd-SrtB_ΔN26_, energy minimization and MD simulations were conducted by gradually releasing the restraints on PPKTG, first on the side chains and then on the entire peptide, whereas the Cd-SrtB_ΔN26_ residues and crystal waters were restrained to their atomic positions in the Cd-SrtB_ΔN26_ structure at all times during the simulations.

**Figure 3:**
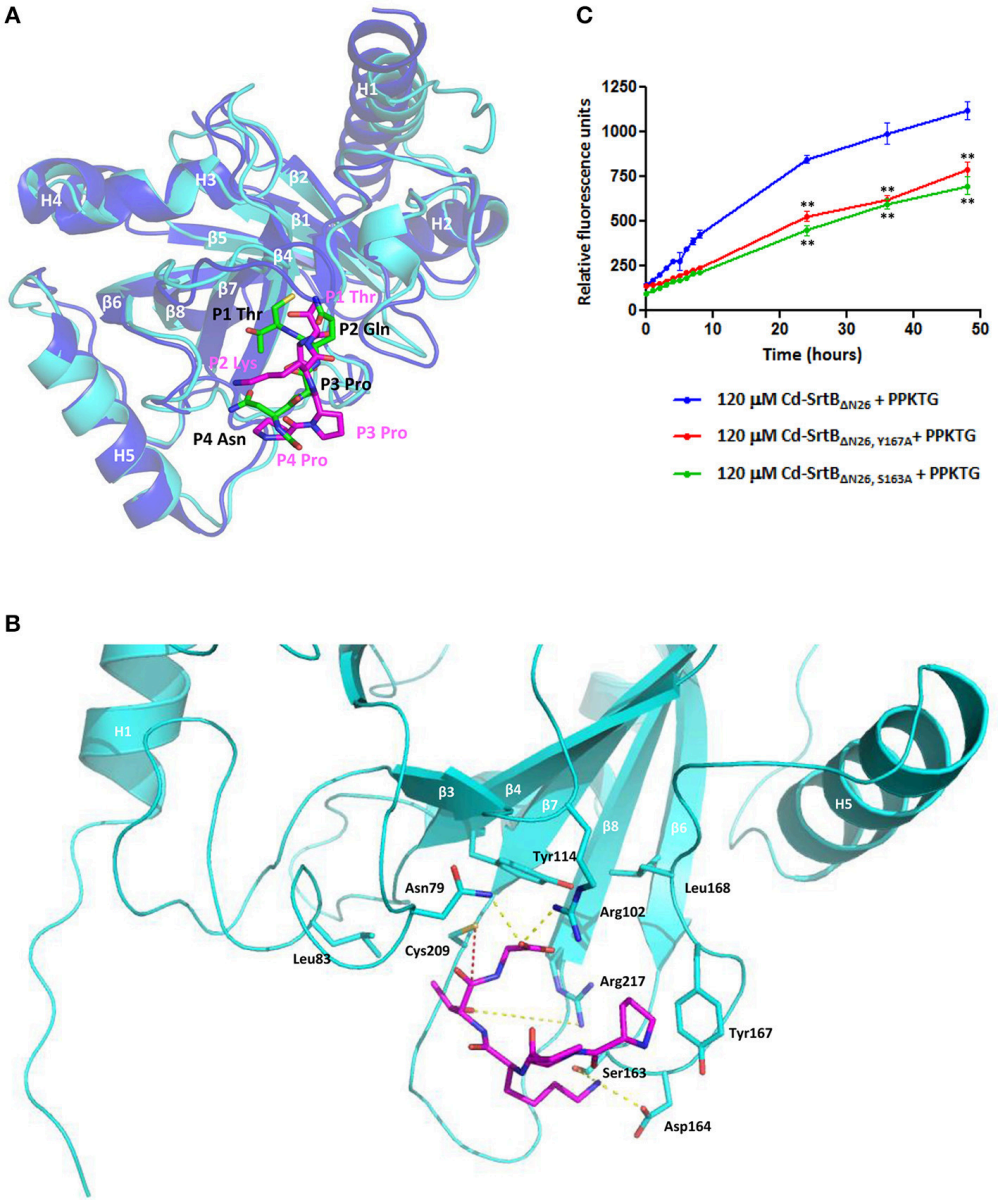
**A computational model of the Cd-SrtB_ΔN26_–PPKTG complex. (A)** Superimposed structures of the Sa-SrtB–NPQT^*^ and Cd-SrtB_ΔN26_–PPKTG models. Blue, Sa-SrtB. Cyan, Cd-SrtB_ΔN26_. NPQT^*^ and PPKTG are presented in green and magenta, respectively. **(B)** The atomic interactions between PPKTG and Cd-SrtB_ΔN26_ in the structural model. The hydrogen bond is colored in yellow. The distance from the sulfhydryl group of Cys209 to the carbonyl carbon of P1 Thr is represented in red. **(C)** Catalytic activity of Cd-SrtB_ΔN26_ with mutation monitored using the FRET-based assay. PPKTG was incubated with recombinant purified SrtB_ΔN26_, SrtB_ΔN26, Y167A_, and SrtB_ΔN26, S163A_. The increase in the relative fluorescence signal was observed when PPKTG was cleaved by Cd-SrtB_ΔN26_. The enzymatic cleavage of Cd-SrtB_ΔN26_ was inhibited with Y167A and S163A mutations.

The results from the computational modeling and unrestrained MD simulations suggest that PPKTG stays in the active site, forming a L-shape with a bend toward the N-terminus, resembling the structure of Sa-SrtB–NPQT^*^ complex (Jacobitz et al., 2014) (Figure 3A). The sulfhydryl group of Cys209 is 5.0 Å from the carbonyl carbon of the threonine residue at the P1 position (Schechter and Berger, 1967), which is slightly further apart as compared with that of NPQT^*^ in Sa-SrtB (Figure 3B). The side chain of Arg217 is hydrogen bonded to the hydroxyl oxygen of P1 Thr. The P2 Lys forms a hydrogen bond with Ser163 and salt-bridge interactions with Asp164. Moreover, the prolyl ring of P4 Pro noncovalently interacts with the aromatic ring of Tyr167. To assess whether Ser163 and Tyr167 are involved in substrate interactions as predicted in the structural model, two mutants that replace Ser163 and Tyr167 with alanine were generated in Cd-SrtB_ΔN26_ by site-directed mutagenesis. By performing the FRET-based assay, we observed that the cleavage activity of mutants Cd-SrtB_ΔN26, S163A_ and Cd-SrtB_ΔN26, Y167A_ was substantially reduced compared to wild-type Cd-SrtB_ΔN26_ (Figure 3C). The results indicate that the alanine substitution of Ser163 and Tyr167 did affect the interactions between Cd-SrtB_ΔN26_ and PPTKG, resulting in the reduced florescence signals.

### Specificity determinants of substrate peptides

**Figure 4:**
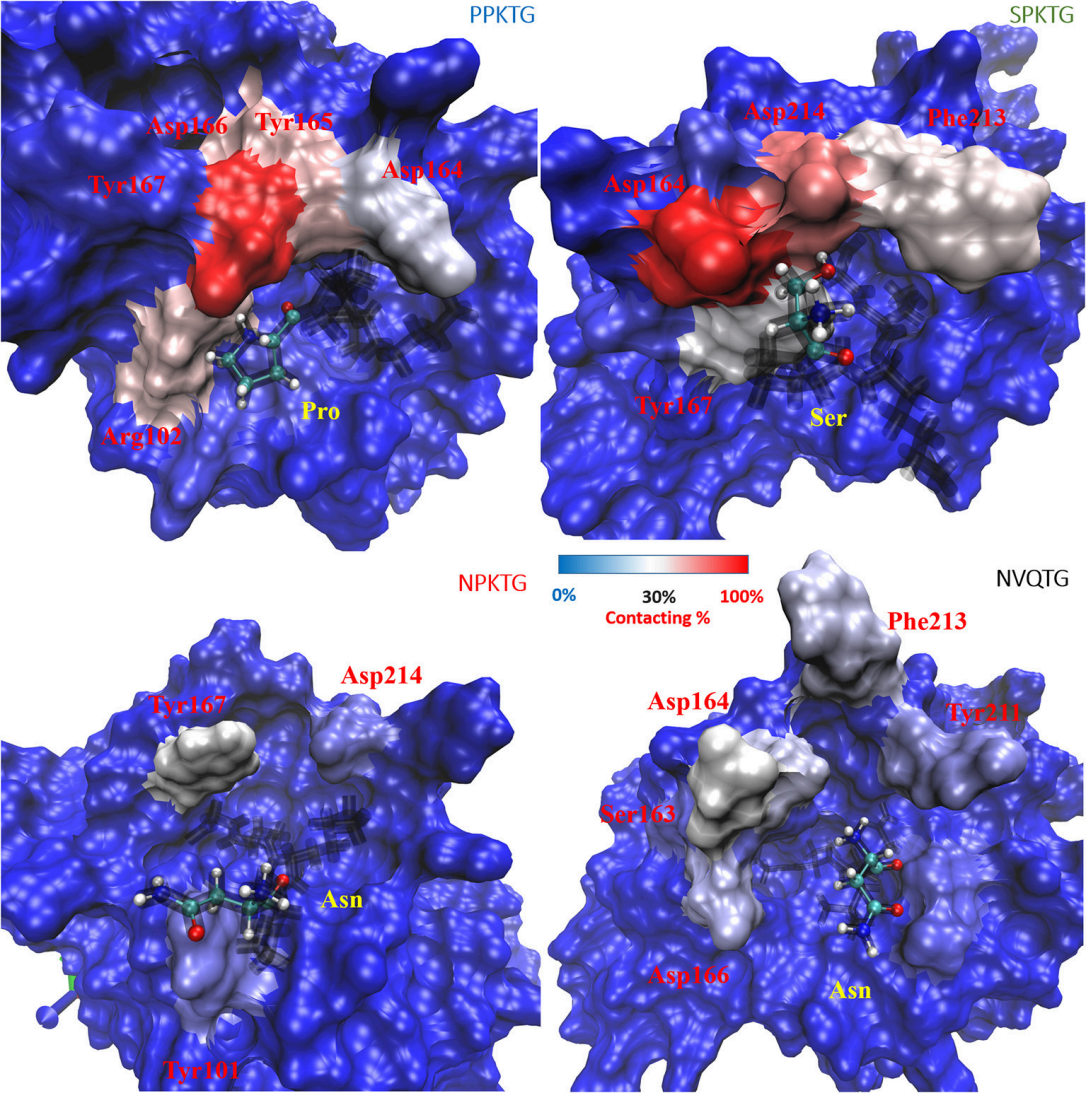
**Contact frequency (to the P4 residue of substrate peptides) for residues in Cd-SrtB_ΔN26_**. Cd-SrtB_ΔN26_ are shown in surface representation and opaque for the cases of Cd-SrtB_ΔN26_–PPKTG (upper-left), Cd-SrtB_ΔN26_–SPKTG (upper-right), Cd-SrtB_ΔN26_–NPKTG (lower-left) and Cd-SrtB_ΔN26_–NVQTG (lower-right). Different peptides are shown as black line, with the P4 residue labeled by ball-and-stick in CPK. The increased contact frequency for the P4 residues in different peptides is colored from blue to red for the purpose of easier visualization.

**Figure 5:**
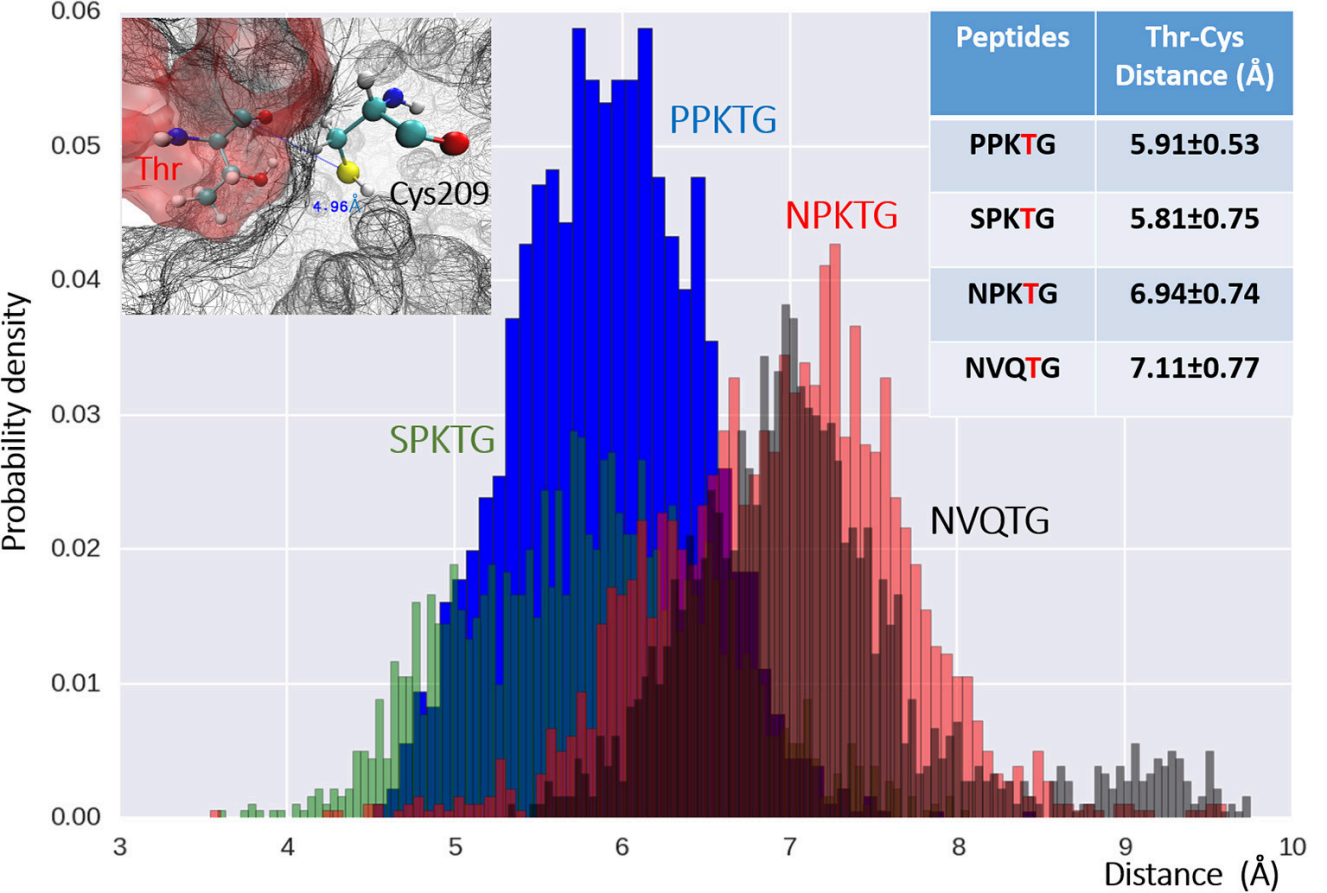
**The distribution of distances between Cys209 (on SrtB_ΔN26_) and Thr4 (on the peptides) for four examined peptides**. Distances between the catalytic Cys209 in Cd-SrtB_ΔN26_ (upper-left) and Thr4, the P4 residue in the peptides of Cd-SrtB_ΔN26_–PPKTG (blue), Cd- SrtB_ΔN26_–SPKTG (green), Cd-SrtB_ΔN26_–NPKTG (red) and Cd-SrtB_ΔN26_–NVQTG (black) of all the snapshots in simulations, are plotted in histogram. Mean distance and deviation for each peptide are provided in the table (upper-right). X-axis is the distance from the sulfur atom of Cys209 to the carboxyl carbon of the Thr in the peptides.

To have a better understanding of specific recognition of the substrate peptide PPKTG by Cd-SrtB, we also constructed models of Cd-SrtB_ΔN26_–SPKTG, Cd-SrtB_ΔN26_–NPKTG, and Cd-SrtB_ΔN26_–NVQTG complexes in the same way as constructing model of Cd-SrtB_ΔN26_–PPKTG complex. Subsequently, we performed molecular dynamics (MD) simulations to analyze the residues in Cd-SrtB_ΔN26_ and the contact frequency of those residues with different substrate peptides (Supplementary Figure 5). It is observed that the P4 residues in PPKTG and SPKTG are stabilized by residues of Cd-SrtB_ΔN26_ located in binding pocket (Tyr101–Arg102, Ser163–Tyr167 for PPKTG, and Asp164–167, Phe213–Asp214 for SPKTG) (Figure 4). Our results show that the P4 residue of PPKTG in Cd-SrtB_ΔN26_–PPKTG complex interacts with Asp166 for about 80% of the simulations time (10 ns) and forms hydrogen bonds with Asp166; while the P4 residues in NPKTG and NVQTG do not specifically interact with any residue in Cd-SrtB_ΔN26_ (Figure 4 and Supplementary Figure 5). Moreover, we assumed that a peptide would be subjected to a conformation that allows Cd-SrtB to achieve a better catalytic efficiency if the distance (DIS_Cys-Thr_) between the sulfhydryl group of cysteine residue in Cd-SrtB and the carboxyl carbon of threonine residue in peptide is relatively short (Donahue et al., 2014; Chambers et al., 2015). We therefore examined distance distributions for four peptides of interest throughout 10 ns simulations. The distances are found to be 5.91 ± 0.53 Å, 5.81 ± 0.75 Å, 6.94 ± 0.74 Å, and 7.11 ± 0.77 Å for Cd-SrtB_ΔN26_–PPKTG, Cd-SrtB_ΔN26_–SPKTG, Cd-SrtB_ΔN26_–NPKTG, and Cd-SrtB_ΔN26_–NVQTG, respectively (Figure 5). To further explore the role of the P4 residues of peptides in substrate specificity, we calculated their root-mean square fluctuations (RMSFs) of the peptides in Cd-SrtB_ΔN26_–PPKTG, Cd-SrtB_ΔN26_–SPKTG, Cd-SrtB_ΔN26_–NPKTG, and Cd-SrtB_ΔN26_–NVQTG. The RMSFs are 0.19, 0.38, 0.48, and 0.58 Å for PPKTG SPKTG, NPKTG and NVQTG, respectively. The higher stability of P4 residues in (P/S)PKTG seen in our dynamic simulations correlates to the shorter DIS_Cys-Thr_ and previously shown higher reaction activity (Donahue et al., 2014; Chambers et al., 2015).

## Discussion

In this work, we have presented the crystal structure of the catalytically active SrtB from *C. difficile* and provided a plausible interaction scheme to understand how SrtB recognizes the unique (S/P)PXTG motif.

The P4 residue of the sortase substrate is likely to be the specificity determinant. Based on our computational model of Cd-SrtB_ΔN26_–PPKTG complex, we are able to identify that the hydrophobic residue Tyr167 in Cd-SrtB_ΔN26_ forms specific interaction with P4 Pro in PPKTG, confirmed by site-directed mutagenesis and FRET-based assay (Figure 3). This hydrophobic interaction between sortase and substrate has not been seen in the current available crystal structures and may be unique to the Cd-SrtB_ΔN26_–PPKTG complex. In the structure of Sa-SrtB–NPQT^*^ complex, P4 Asn in NPQT^*^ is hydrogen bonded to the carbonyl backbone of Thr177 within the β6/β7 loop in Sa-SrtB (Jacobitz et al., 2014). The residue Thr177 in Sa-SrtB is structurally equivalent to that of Ser163 in Cd-SrtB_ΔN26_. However, structural superposition shows Ser163 in Cd-SrtB_ΔN26_ is too far away to interact with P4 Asn in Sa-SrtB–NPQT^*^. Moreover, we also identified that Ser163 interacts with P2 Lys in PPKTG. As Tyr167A and Ser163A mutants exhibited reduced hydrolytic activity (Figure 3), we concluded that these two residues play important roles in specific substrate-binding and that the abolishment of the specific interactions affects the cleavage activity by Cd-SrtB_ΔN26_. Taken together, the structural analyses have provided partial explanation why Cd-SrtB_ΔN26_ does not recognize the NPQTN sorting signal. However, the actual crystal structure of Cd-SrtB–(S/P)PKT^*^ is required to disclose the atomic interactions of the complex.

The simulation studies on the structural models of Cd-SrtB_ΔN26_–PPKTG, Cd-SrtB_ΔN26_–SPKTG, Cd-SrtB_ΔN26_–NPKTG, and Cd-SrtB_ΔN26_–NVQTG complexes suggest that the stability of P4 residue may have an effect on the position P1 residue and DIS_Cys-Thr_ (Figures 4, 5). It seems reasonable to imply that the P4 Pro in PPKTG plays a role in configuring the substrate peptide to a preferred conformation, permitting Cd-SrtB to perform a more efficient cleavage. Furthermore, PPKTG and SPKTG that have a better Cd-SrtB_ΔN26_ hydrolytic activity than NPKTG and NVQTG peptides (Donahue et al., 2014; Chambers et al., 2015) are found to have comparatively high contacting frequency with Cd-SrtB_ΔN26_ via their P4 residue and a shorter DIS_Cys-Thr_ throughout the simulations. It suggests the stabilization of P4 residue by surrounding loops near the active site can refrain the mobility of substrate peptides and therefore result in a shorter DIS_Cys-Thr_ prompted for catalysis. The specificity determinant that associates with P4-led peptide conformation provides a molecular basis for specific recognition of PPKTG by Cd-SrtB.

## Author contributions

LY, IH, and SW conceived and designed the experiments. JY, CF, YL, JC, and YC performed he experiments. JY, YL, YH, JN, LY, IH, and SW analyzed the data. JY, CF, YL, LY, IH, and SW prepared the manuscript.

### Conflict of interest statement

The authors declare that the research was conducted in the absence of any commercial or financial relationships that could be construed as a potential conflict of interest.
