# Supplementary material for: Structural Insights into Substrate Recognition by Clostridium difficile Sortase
Source: Front Cell Infect Microbiol. 2016 Nov 22;6:160. doi: 10.3389/fcimb.2016.00160 (PMC5118464; doi:10.3389/fcimb.2016.00160)
Supplement: Supplementary file 1 [file DataSheet1.DOCX]

Supplementary Material

Structural insights into substrate recognition by *Clostridium difficile* sortase

Jui-Chieh Yin^§^, Chun-Hsien Fei^§^, Yen-Chen Lo^§^, Yu-Yuan Hsiao, Jyun-Cyuan Chang, Jay C. Nix, Yuan-Yu Chang, Lee-Wei Yang^*^, I-Hsiu Huang^*^, Shuying Wang^*^

Correspondence:

Lee-Wei Yang: [lwyang@life.nthu.edu.tw](mailto:lwyang@life.nthu.edu.tw)

I-Hsiu Huang: [ihsiuhuang@mail.ncku.edu.tw](mailto:ihsiuhuang@mail.ncku.edu.tw)

Shuying Wang: [sswang23@mail.ncku.edu.tw](mailto:sswang23@mail.ncku.edu.tw)


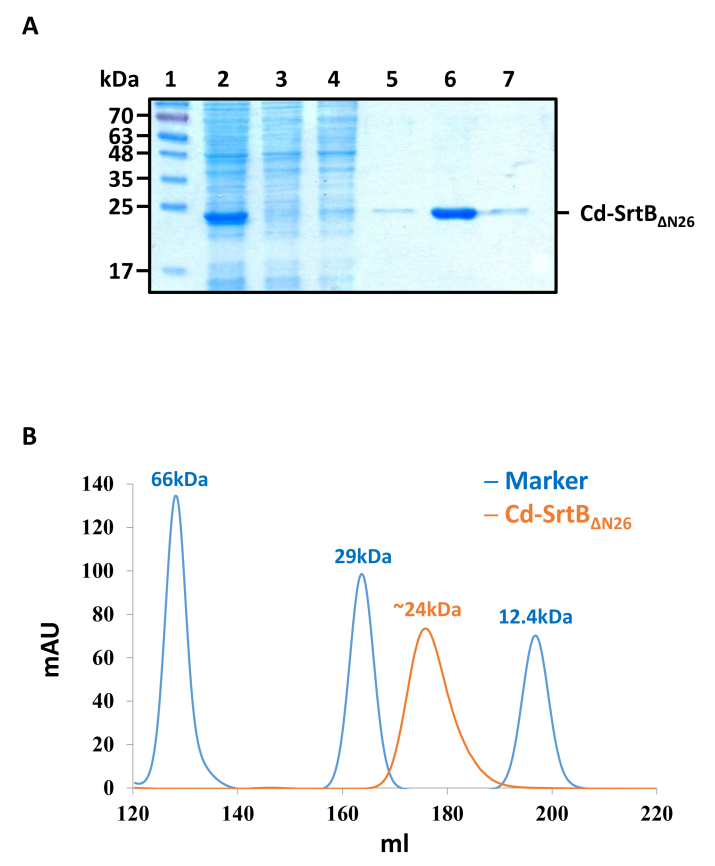


**Supplementary Figure 1.** **Expression and purification of Cd-SrtB_ΔN26_.** (A) SDS-PAGE of the recombinant purified wild-type Cd**-**SrtB_ΔN26_. Lane 1, protein marker; lane 2, soluble fraction of the cell lysate; lane 3, flow-through of unbound proteins; lane 4, washed proteins; lanes 5–7 purified SrtB_ΔN26_ eluted from an Ni^2+^-NTA column. (B) Size exclusion chromatography profile of the purified SrtB_ΔN26_. Blue line: standard molecular weight marker: BSA, 66 kDa; carbonic anhydrase, 29 kDa; cytochrome c, 12.4 kDa. Orange line: purified SrtB_ΔN26_.


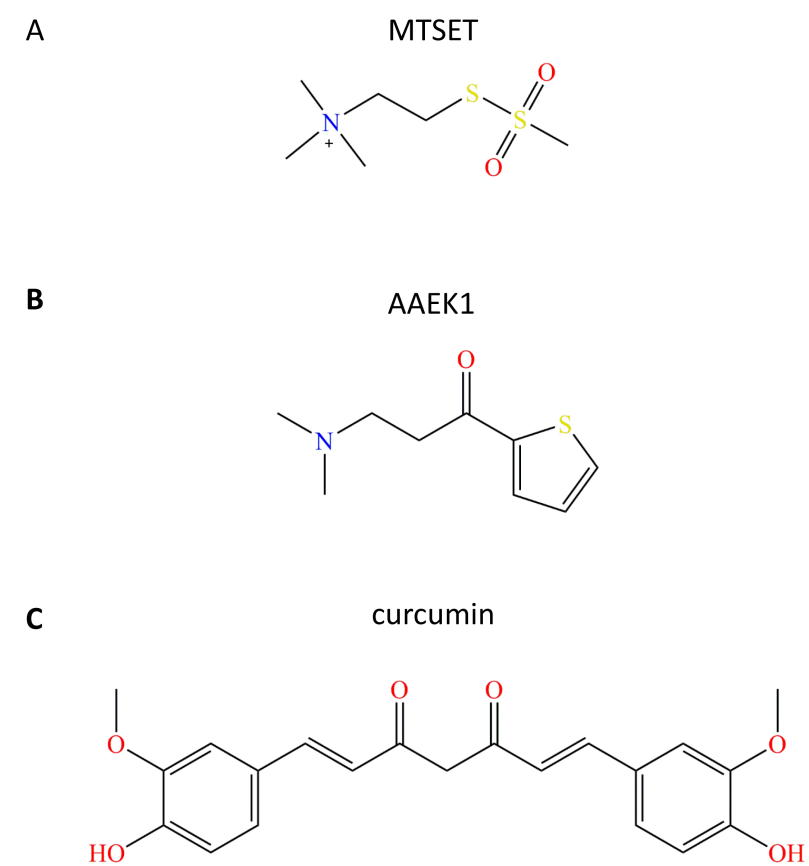


**Supplementary Figure 2.** **Chemical structures of SrtB inhibitors.** (A) MTSET, (B) AAEK1, and (C) curcumin.


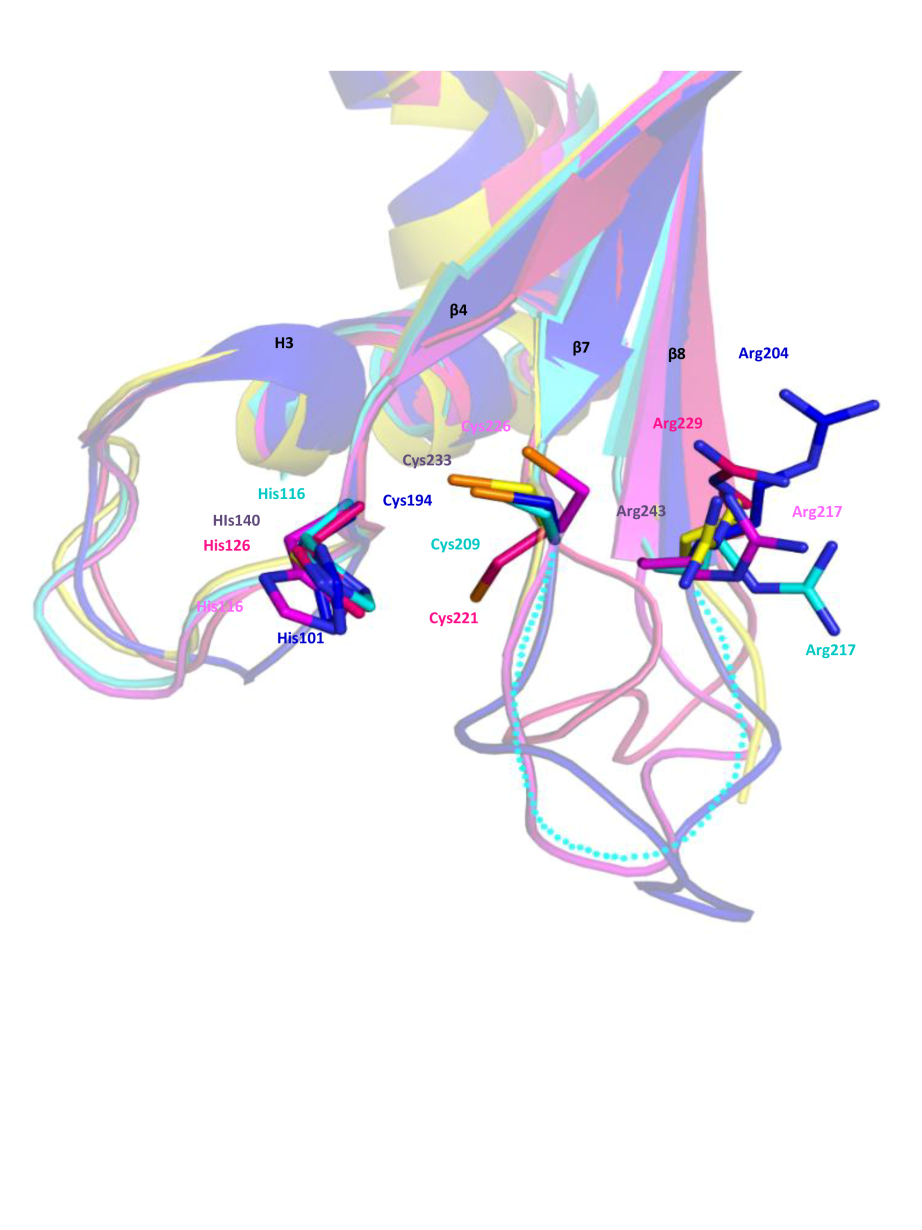


**Supplementary Figure 3.** **Structural similarity of the SrtB structures.** Structural superimposition of the active sites of Cd-SrtB_ΔN26_ (cyan) with that of *C. difficile* SrtB_ΔN32,C226A_ (magenta), *B. anthracis* SrtB (yellow), Sa-SrtB (blue), and *Streptococcus pyogenes* SrtB (pink).

**
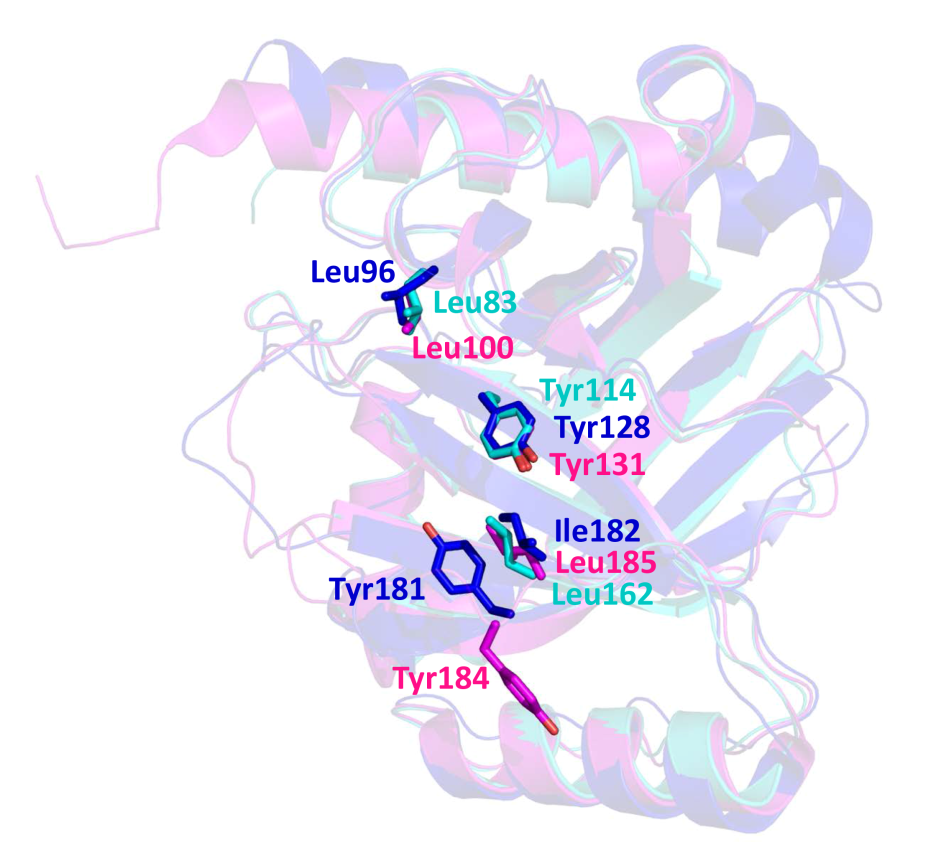
**

**Supplementary Figure 4.** **Superimposed hydrophobic residues from structures of Sa-SrtB–NPQT*, Cd-SrtB_ΔN26_, and Cd-SrtB_ΔN32,C226A_.** Residues of Leu96, Tyr128, Tyr181 and Ile182 from Sa-SrtB–NPQT* (PDB 4LFD) are presented in blue. Residues of Leu83, Tyr114, and Leu162 from Cd-SrtB_ΔN26_ (PDB 5GYJ) are presented in cyan. Residues of Leu100, Tyr131, Tyr184 and Leu185 from Cd-SrtB_ΔN32,C226A_ (PDB 4UX7) are presented in magenta.


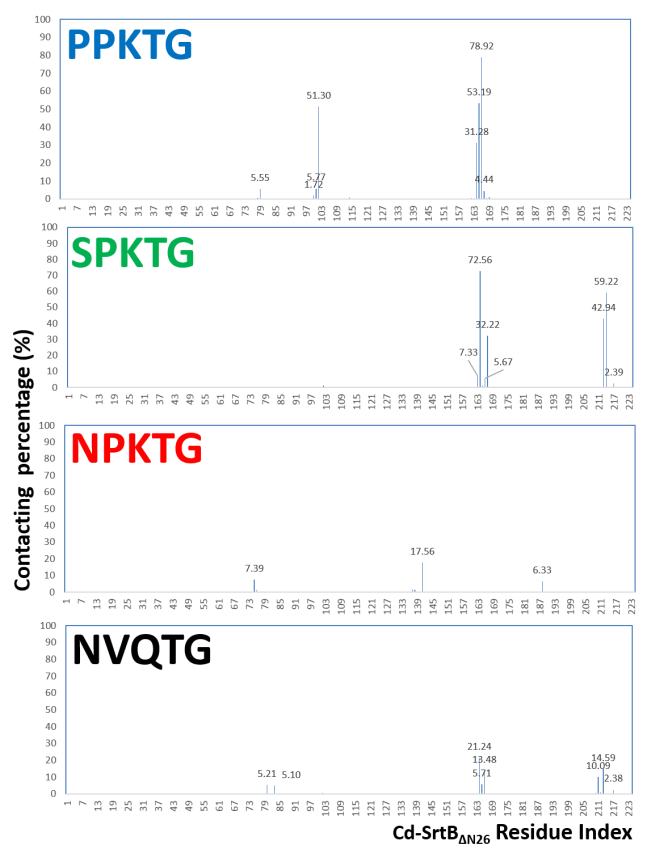


**Supplementary Figure 5.** **Contacting Residues (from Cd-SrtB) interacting with the P4 residue (the 1^st^ residue from the N-terminus in the peptide) of different substrate peptides.**Contacting residues are defined as those from Cd-SrtB that situate within 4.0 Å from the P4 residue of different substrate peptides (A) PPKTG, (B) SPKTG, (C) NPKTG and (D) NVQTG. Contacting frequency is calculated as the frames where a specific contact is observed divided by the total number of frames in the simulations. In the figure, Y-axis  represents the percentage of Cd-SrtB_ΔN26_ residues contacting with the 1^st^ residue in the peptide (100% means a given residue contacts with the 1^st^ residue in the peptide in every frame of the simulation), X-axis is the residue index of Cd-SrtB_ΔN26._
